# Phytochemical Evaluation, Antimicrobial Activity, and Determination of Bioactive Components from Leaves of *Aegle marmelos*

**DOI:** 10.1155/2014/497606

**Published:** 2014-05-11

**Authors:** Farina Mujeeb, Preeti Bajpai, Neelam Pathak

**Affiliations:** Department of Biosciences, Integral University, Kursi Road, Lucknow 226026, India

## Abstract

The therapeutic value of *Aegle marmelos* Correa (Rutaceae), commonly known as ‘‘Bael,” has been recognized as a component of traditional medication for the treatment of various human ailments. The plant, though, being highly explored, still lacks sufficient evidences for the best variety possessing the highest degree of medicinal values. The present study is focused on phytochemical screening of aqueous and methanolic leaf extracts of 18 varieties/accessions of *A. marmelos*. The crude extracts of *A. marmelos* revealed the presence of several biologically active phytochemicals with the highest quantity of alkaloids, flavonoids, and phenols in Pant Aparna variety. The antibacterial efficacy was investigated against pathogenic bacterial strains and the highest inhibitory activity of aqueous extract was obtained against *S. epidermidis*, whereas methanolic extract was found to be most potent against *S. aureus* at 40 mg/mL concentration. However, in aqueous : ethanol, the best results were observed against *E. aerogenes* followed by *K. pneumonia* and *S. epidermidis*. The MIC of aqueous and methanol extract of *Aegle marmelos* ranged from 10 mg/mL to 40 mg/mL whereas in aqueous : ethanol it ranged between 40 mg/mL and 160 mg/mL. The GC-MS analysis revealed the presence of many bioactive compounds such as flavonoids, alcohols, aldehydes, aromatic compounds, fatty acid methyl esters, terpenoids, phenolics, and steroids that can be postulated for antibacterial activity.

## 1. Introduction

India is widely known as the botanical garden of the world since it is the largest producer of medicinal herbs [1]. Medicinal plants act as an indigenous source of new compounds possessing therapeutic value and can also be used in drug development. 80% of the population of developing countries depend on traditional medicines, mostly natural plant products, for their primary health care needs as estimated by WHO [2]. Because of the growing recognition of natural products the demand for medicinal plants has been increasing all over the world. They have minimal toxicity, are cost effective and pharmacologically active, and provide an easy remedy for many human ailments as compared to the synthetic drugs which are a subject of adulteration and side effects [3]. The alarming increase in the rate of infection by antibiotic-resistant microorganisms has urged scientists to search for compounds which have potential antimicrobial activity [4]. The ability to synthesize compounds by secondary metabolism possessing antimicrobial potential makes plants an invaluable source of pharmaceutical and therapeutic products [5]. The effectiveness of plant extracts on microorganism has been studied worldwide [6–9].

Bael (*Aegle marmelos*) has been known to be one of the most important medicinal plants of India since Charak (1500 B.C) [10]. More than 100 phytochemical compounds have been isolated from various parts of the plant, namely phenols, flavonoids, alkaloids, cardiac glycosides, saponins, terpenoids, steroids, and tannins. These compounds are well known to possess biological and pharmacological activity against various chronic diseases such as cancer and cardiovascular and gastrointestinal disorders [11–14]. Antioxidant, antiulcer, antidiabetic, anticancer, antihyperlipidaemic, anti-inflammatory, antimicrobial, antispermatogenic effects have also been reported on various animal models by the crude extracts of this plant [14–22]. Every part of* Aegle marmelos* plant such as its fruits, stem, bark, and leaves possesses medicinal property and is used for treating various eye and skin infections [23]. Leaf is considered to be one of the highest accumulatory parts of the plant containing bioactive compounds which are synthesized as secondary metabolites [24]. The present study was, therefore, aimed at evaluating the phytochemical potential and antibacterial activity of* Aegle marmelos* aqueous and methanolic leaf extracts.

## 2. Materials and Methods

### 2.1. Collection and Identification of Plant Material

The fresh leaves of* Aegle marmelos* from 18 varieties/accessions were collected from the orchard of Narendra Deva University of Agriculture and Technology, Kumarganj, Faizabad, India. The taxonomy of the plant was authenticated.

### 2.2. Extract Preparation

The leaves of the plant were properly washed in tap water and rinsed in distilled water. The rinsed leaves were hot air-dried for 3 days. The dried leaves of each plant were pulverized using pestle mortar to obtain a powdered form which was stored in airtight glass containers at 4°C until used. 10 g of powdered sample was soaked in distilled water and methanol (200 mL and 100 mL) separately for 12 hrs at room temperature. The extracts were then filtered and concentrated to a final volume of 50 mL and subjected to phytochemical analysis.

For antibacterial screening the leaf extracts were prepared from Pant Aparna variety by following the protocol of Harborne [25] in Soxhlet apparatus using 180 mL of distilled water and methanol. For aqueous: ethanol, 50 : 130 volume of distilled water and ethanol were taken. Every extraction was carried out for 24 hrs and the extract was then dried, weighed, and stored in refrigerator at 4°C.

### 2.3. Phytochemical Analysis

Qualitative phytochemical analyses of both the extracts were performed by following the protocol of Adetuyi and Popoola [26], Trease and Evans [27], and Sofowora [28].

*Tannins.* 200 mg of plant material was boiled in 10 mL distilled water and few drops of FeCl_3_ were added to the filtrate; a blue-black precipitate indicated the presence of Tannins.

*Alkaloids.* 200 mg plant material was boiled in 10 mL methanol and filtered. 1% HCl was added followed by 6 drops of Dragendorff reagent, and brownish-red precipitate was taken as evidence for the presence of alkaloids.

*Saponins (Frothing test).* 5 mL distilled water was added to 200 mg plant material. 0.5 mL filtrate was diluted to 5 mL with distilled water and shaken vigorously for 2 minutes. Formation of stable foam indicates the presence of saponins.

*Cardiac Glycosides (Keller-Kiliani test).* 2 mL filtrate was treated with 1 mL glacial acetic acid containing few drops of FeCl_3_.Conc. H_2_SO_4_ was added to the above mixture giving green-blue colour depicting the positive results for presence of cardiac glycosides.

*Steroids (Liebermann-Burchard reaction).* 200 mg plant material was added in 10 mL chloroform. Acetic anhydride was added in the ratio of 1 : 1 which resulted into the formation of blue-green ring pointing towards the presence of steroids.

*Terpenoids (Salkowski test).* To 200 mg plant material 2 mL of chloroform (CHCl_3_) and 3 mL of concentrated sulphuric acid (H_2_SO_4_) were carefully added. A reddish brown colouration signified the presence of terpenoids.

*Flavonoids.* To the aqueous filtrate 5 mL of dilute ammonia solution was added, followed by concentrated H_2_SO_4_. A yellow colouration indicated the presence of flavonoids.

*Phlobatannins.* The deposition of a red precipitate denoted the presence of phlobatannins when 200 mg of plant material was dissolved in 10 mL of aqueous extract and few drops of 1% HCl were added in the boiling tube.

*Anthraquinones.* 500 mg of dried plant leaves were boiled in 10% HCl for 5 mins and filtrate was allowed to cool. Equal volume of CHCl_3_ with few drops of 10% NH_3_ was added to 2 mL filtrate. The formation of rose-pink colour implies the presence of Anthraquinones.

*Reducing Sugars.* To the 10 mL of aqueous extract a few drops of Fehling's solution A and B were added; an orange red precipitate suggests the presence of reducing sugars.

### 2.4. Quantitative Estimation of Phytochemicals

#### 2.4.1. Determination of Alkaloids

Alkaloids content was measured by following the protocol described by Harborne [29]. A suspension was prepared by dispersing 5 gm of the dried leaves in 10% acetic acid solution in ethanol and kept at 28°C for 4 hrs which was further filtered through Whatman Number 42. Thereafter alkaloid was precipitated by concentrating the filtrate to one quarter of its original volume and drops of conc. aqueous NH_4_OH were added. Finally the precipitate was washed with 1% ammonia solution and dried at 80°C in the oven. The content of alkaloid was calculated and expressed as mg/gm of sample.

#### 2.4.2. Determination of Flavonoids

The flavonoids content was also determined by Harborne [29] method. Briefly, 5 gm of leaves was boiled in 2 M HCl for 30 mins under reflux and filtered after cooling. Equal volume of ethyl acetate was then added drop wise in filtrate. The weight of precipitated flavonoid was determined and reported as mg/g.

#### 2.4.3. Determination of Tannins

The quantitative estimation of tannins was performed by the method of Swain [30] with minor modifications in our lab. Finely powdered leaves of* Aegle marmelos* were kept in a beaker containing 20 mL of 50% methanol covered with parafilm and then heated at 80°C in water bath for 1 hr with continuous stirring. The extract was quantitatively filtered using a double layered Whatman Number 1 filter paper and rinsed by 50% methanol. 1 mL of sample extract was treated with 20 mL distilled water, 2.5 mL Folin-Denis reagent, and 10 mL of 17% Na_2_CO_3_ for the development of a bluish-green colour and was allowed to stand for 20 minutes. The absorbance was measured at 760 nm and amount of tannin was calculated by comparing it with standard curve prepared in the range of 0–10 ppm.

#### 2.4.4. Determination of Saponins

Saponin analysis was performed according to the method described by Brunner [31]. 100 mL Isobutyl alcohol was added to 1 g of finely powdered sample and stirred for 5 hrs. 20 mL of 40% saturated solution of Magnesium carbonate was added to the mixture and filtered. 2 mL of 5% FeCl_3_ solution and 50 mL volume of distilled water was added to 1 mL of colourless solution and kept for 30 min for colour (blood red) development. The absorbance of the samples along with the standard were read at 380 nm and calculated in mg/gm. Standard saponin solution was prepared in the reference range of 0–10 ppm.

#### 2.4.5. Determination of Total Phenols

Five grams of the powdered leaves was boiled with 50 mL of ether for 15 mins and distributed in the ratio 1 : 2 (extract : distilled water). 2 mL of ammonium hydroxide followed with 5 mL of pentanol was added to it and incubated at the room temperature for 30 minutes. The absorbance was read at 505 nm as described by Obodoni and Ochuko [32].

### 2.5. Procuring of Bacterial Strain

Pure cultures of five test organisms, namely,* S. aureus* (NCIM 2079),* S. epidermidis* (NCIM 2493),* B. cereus* (NCIM 2156),* E. aerogenes* (NCIM 5139), and* K. pneumoniae* (NCIM 2957), were procured from National Chemical Laboratory, Pune. Stock cultures were maintained at 4°C on agar slants of nutrient media. Active cultures for experiment were prepared by transferring a loop full of microorganism from the stock cultures to 50 mL of sterile nutrient broth.

### 2.6. Antibacterial Activity

The extracts mentioned above were tested against five pathogenic bacterial strains, three gram-positive bacteria (*B. cereus*,* S. epidermidis*, and* S. aureus*), and two gram-negative bacteria (*E. aerogens*,* K. pneumoniae*). Antibacterial screening was done using agar well diffusion method [33]. For this 20 mL of sterile Mueller-Hinton Agar (Hi-media) was poured in sterile autoclaved petri plates. After solidification, the sterile cotton swab was dipped into the bacterial culture. The entire agar surface of each plate was evenly inoculated by swabbing. The seven uniform wells were prepared with the help of sterile 6 mm diameter cork-borer. Each well was filled with the various concentrations of both the aqueous and methanol extract (10, 20, 25, 30, and 40 mg/mL), respectively, whereas, in case of aqueous: ethanol, (40, 80, 100, and 120 mg/mL) concentrations were used and allowed for diffusion for 45 minutes. The plates were then incubated at 37°C for 24 hrs. Triplicate plates were prepared for each treatment and the average zone of inhibition excluding well was recorded. 9% DMSO was used as negative control. Turbidity of bacterial culture was maintained up to 1 × 10^8^ CFU/mL. The antibacterial potential of extracts was compared with standard antibiotic Ampicillin (10 *μ*g/disc) with paper disc (Hi-media) method.

### 2.7. Gas Chromatography Mass Spectrometry Analysis

The gas chromatography-mass spectrometry (GC-MS) analysis of methanolic extract of leaves of* Aegle marmelos* (var. Pant Aparna)was performed using a GC-MS (Model; QP 2010 Plus, Shimadzu, Tokyo, Japan) equipped with a VF-5 ms fused silica capillary column of 30 m length, 0.25 mm diameter, and 0.25 *μ*m film thickness. The column oven temperature was programmed from 80°C to 310°C for 2°C min^−1^. Ionization of the sample components was performed in electron impact mode (EI, 70 eV). The temperature of the injector was fixed to 270°C and detector to 230°C. Helium (99.9995% purity) was the carrier gas fixed with a flow rate of 1.21 mL min^−1^. The mass range from 40–650* m*/*z* was scanned at a rate of 3.0 scans/s. 2.0 *μ*L of the methanolic extract of* Aegle marmelos* was injected with a Hamilton syringe to the GC-MS manually for total ion chromatographic analysis in split injection technique. Total running time of GC-MS was 56 min. The relative percentage of each extract constituent was expressed as percentage with peak area normalization.

The bioactive compounds of methanol extract were identified by comparing their retention indices and patterns of mass spectra with reference to Wiley Registry of Mass Spectral Data's, New York (Wiley 8) and Fatty Acid Methyl Esters Library version 1.0 (FAME library) sources.

## 3. Result and Discussion

### 3.1. Phytochemical Profiling

The present study was carried on aqueous and methanolic extracts of* Aegle marmelos* to investigate the presence of medicinally important phytochemicals in the leaves of different varieties/accessions. Both the extracts revealed the presence of various phytochemicals such as tannins, saponins, flavonoids, alkaloids, terpenoids, carotenoids, cardiac glycosides, and reducing sugars in all the varieties and accessions while phlobatannins and anthocyanins were absent (Table 1). The presence of different phytochemicals and the antimicrobial activity of ethanolic, petroleum ether, chloroform, and methanolic extract of a single unidentified variety of* Aegle marmelos *have been previously reported [34, 35]; however, our study is first ever report to the best of our knowledge on qualitative and quantitative comparative analysis of various varieties/accessions available in India. The findings conclude that amongst 18 varieties and accessions, the variety Pant Aparna was found to be the best. The antibacterial activity of this variety using methanol, aqueous, and aqueous: ethanol as the solvent has been investigated. The activity was further confirmed by performing GC-MS which revealed the presence of different phytochemicals.

The quantitative phytochemical estimation specifies that the leaves of all varieties contain a significant amount of alkaloid, flavonoids, phenolic, saponins, and tannin content. However the variety called Pant Aparna contains the highest amount of alkaloids, flavonoids, and phenol (Table 2). The alkaloids content was quantitatively estimated and was found in the range of 3.78 ± 0.15–16.08 ± 0.05 mg/gm in different varieties of Bael leaves but the maximum content was observed in Pant Aparna (16.08 ± 0.05 mg/gm). Similarly all the varieties exhibited good quantity of flavonoids and phenols starting from 10.4 ± 0.047 mg/mL and 5.8 ± 0.085 mg/mL, respectively, in variety NB-1, reaching up to 63.9 ± 0.061 mg/mL and 29.4 ± 0.004 in Pant Aparna. Earlier report on quantitative yield also revealed that* Aegle marmelos* contained highest quantity of alkaloids, flavonoids, and tannins as compared to other medicinal plants [36]. The highest content of saponins was found in variety AM-7 (13.40 ± 0.30) followed by Pant Aparna and AM-6. Among all the varieties analysed, Pant Aparna was found to be the most promising one, which prompted us to project Pant Aparna for further studies.

### 3.2. Antibacterial Study

The pharmacological action of the plant cannot be ascertained by the result of phytochemical studies only. Thus the antibacterial activity against pathogenic bacteria was also evaluated. The present investigation shows the efficacy of all the extracts against the selected pathogenic bacteria (Tables 3, 4, and 5).

The aqueous extract showed highest antibacterial activity against* S. epidermidis* (14.3 mm) followed by* S. aureus* (13 mm) and* K. pneumonia *(10.6 mm). The maximum zone of inhibition was observed at the concentration of 40 mg/mL. Varying degree of antibacterial activity by leaf extracts of* Aegle marmelos* against various tested bacterial species has been reported [37]. The presence of the compounds Cuminaldehyde and Eugenol may be responsible for the antibacterial activity of leaf extracts against various bacterial strains [38]. The aqueous, acetone, and petroleum ether extracts of* A. marmelos* were found to be effective against* B. coagulans*,* B. subtilis*,* B. thuringiensis*,* P. aeruginosa*, and* S. aureus* as reported earlier [39]. The dried fruit extract of* Aegle marmelos* showed potential antibacterial activity against* P. aeruginosa*,* S. flexneri*,* E. coli*,* B. subtilis*,* S. epidermidis*, and* S. aureus* [40].

On the other hand, the methanol extract exhibited a much better antibacterial activity as compared to aqueous extract against* K. pneumoniae*,* B. cereus*, and* S. aureus*. The methanol extract was most potent against* S. aureus*, showing the maximum zone of inhibition at the concentration of 40 mg/mL. The highest sensitivity of* S. aureus* may be due to its cell wall structure and outer membrane [41]. No activity was seen against* E. aerogenes*. A few reports have also mentioned significant* in vitro* antimicrobial activity by the methanol extracts of* Aegle marmelos* leaves and flowers [35, 42]. The MIC value of both aqueous and methanol extract was significant at 10 mg/mL; however, for aqueous: ethanol it was 40 mg/mL which is analogous to the MIC values reported using crude extracts of other medicinal plants. The antimicrobial activity of papaya seed extract with MIC value of 11.8 mg/mL was found to be effective against* S. typhi *by Yismaw et al. [43]. In* Zizyphus *sp. MIC values of 25 mg/mL [44] have been reported against* S. aureus*. Similarly, in case of* F. religiosa, *the final MIC values were 2–4-fold higher than initial MIC which ranged from 4.7 to 18.8 mg/mL and from 2.4 to 18.8 mg/mL for resistant and for sensitive isolates, respectively. MIC values (25 mg/mL) were reported by Valsaraj et al. [44] while higher concentration of 150 mg/mL was reported by Ahmad and Beg [45] against* S. aureus. *In yet another study, the antibacterial activity of whole plant of* Borreria hispida *(Linn) was evaluated against various organisms, where the MIC of methanolic extract of* Borreria hispida *was found to range from 250 *μ*g/mL to 50 mg/mL. Moreover higher MIC values ranging from 0.125 to 32 mg/mL were also reported against at least one of the test microorganisms from nine ethnobotanically Indian medicinal plants [46].

The high antibacterial activity in the methanolic extract may be due to the presence of tannins, flavonoids, and terpenoids. These medicinally bioactive components exert antimicrobial action through different mechanism. Tannins cause inhibition in the cell wall synthesis by forming irreversible complexes with prolene rich protein [47]. The saponins have the ability to cause leakage of proteins and certain enzymes from the cell [48]. Terpenoids are responsible for dissolution of the cell wall of microorganism by weakening the membranous tissue [49]. Flavonoids which have been found to be effective antimicrobial substances against a wide array of microorganisms* in vitro* are known to be synthesized in response to microbial infection by plants. They have the ability to complex with extracellular and soluble proteins and to complex with bacterial cell walls [50]. Furthermore, steroids are known for their antibacterial activity specifically associated with membrane lipids and cause leakage from liposomes [51].

In the case of aqueous: ethanol, the maximum antibacterial activity was seen against* E. aerogenes* followed by* S. epidermidis* and* K. pneumonia *(Table 5). At the highest concentration of 160 mg/mL it showed 18.3 mm zone of inhibition. The antibiotic susceptibility showed that among all the bacterial strains* S. aureus* was found to be more susceptible to ampicillin followed by* K. pneumonia*.

### 3.3. GC-MS Analysis

In all, thirty-three compounds were identified from the GC-MS analysis of methanolic extract of Bael leaves exhibiting various phytochemical activities and were predominantly responsible for the antimicrobial activity found in the extract against the pathogenic bacteria. The retention time and percentage peak of various bioactive compounds are presented in Table 6. The major phytoconstituents present in the leaf extract were 1-Dodecanol (4.83), 4H-Pyran-4-one, 2,3-dihydro-3,5-dihydroxy-6-methyl-(1.11), 2,3Dioxabicyclo[2.2.2]oct-5-ene, 1-Methyl-4-(1-Methylethyl)-(Limonene dioxide 1) occupying two peak areas, that is, (0.53) and (0.40), Bicyclo[3.1.1] heptane-2,3-diol, 2,6,6-trimethyl (2,3-Pinanediol)(0.97), 2-Cyclohexen-1-one, 4-hydroxy-3-methyl-6-(1-methylethyl)-(0.63, 0.41, 0.17), Phenol, 2,6-bis(1,1-dimethylethyl)-4-methyl-(BHT) (0.87), Tetradecanoic acid (Myristic acid) (2.00), 2(4H)-Benzofuranone 5,6,7,7A-Tetrahydro-6-hydroxy-4,4,7a-trimethyl,1,3-cyclohexadiene,2-methyl-5-(1methylethyl)-(1-Phellandrene), 2-Propenoic acid, 3-(4-hydroxy-3-methoxyphenyl)-, methyl ester (Cinnamic acid, 4-hydroxy-3-methoxy-, methyl ester), 3,7,11,15-Tetramethyl-2-hexadecen-1-ol, commonly known as phytol; a diterpene has significant antimicrobial properties against many bacterial strains [52]. 9,12,15-Octadecatrienoic acid, methyl ester (Linolenic acid, methyl ester) showing antibacterial and anticandidal activity, 2-Hexadecen-1-ol, 3,7,11,15-Tetramethyl (Phytol isomer) (6.37), Octadecanoic acid (Stearic acid) (4.07), Benzene, 1,2-dimethoxy-4-[[(4 methylphenyl) sulfonyl]methyl (10.76), fatty alcohols such as Ergost-5-en-3-ol, (3.beta.) (campesterol), Stigmasta-5, 22-dien-3-ol, Stigmast-5-en-3-ol, (3.beta.)- may be synergistically responsible for the antimicrobial activity.

The different phyto compounds responsible for bioactivity have been identified and characterized in different medicinal plants. 4H-Pyran-4-one, 2,3-dihydro-3, 5-dihydroxy-6-methyl-, a potent anti-inflammatory and antioxidant compound, possessed antibacterial activity in* Barleria prionitis* (Linn.) rhizome. A long-chain fatty alcohol, 1-Dodecanol, was reported with highest antibacterial activity against* Staphylococcus aureus* [53]. Phenol, 2,6-bis(1,1-dimethylethyl)-4-methyl commonly known as Butylated hydroxytoluene (BHT), an antioxidant, has also demonstrated marked antimicrobial activity inhibiting or decreasing the growth of gram-positive bacteria at a higher degree than the gram-negative bacteria belonging to the family Enterobacteriaceae [54]. Derivatives of cinnamic acid such as esters, amides, acids, and hydrazides have been reported to have antibacterial, antifungal, and antiviral properties [55]. 2(4H)-Benzofuranone, 5,6,7,7a-tetrahydro-4,4,7a-trimethyl, a bioactive compound possessing the properties such as analgesic, antidiabetic, antibacterial, and antifungal, was identified from the methanolic fractions of the* Azadirachta indica* [56]. The extract contained (0.23%) of 1,3-Cyclohexadiene, 2-methyl-5-(1-methylethyl), commonly known as 1-phellandrene. Stigmasterol has been reported earlier as a strong antioxidant having antibacterial activity against multidrug resistant mycobacteria [57, 58].

Several bioactive compounds, namely, Marmin [59] and Marmelosin coumarin derivatives [60] and Aegeline, an alkaloid, have been previously reported from* Aegle marmelos* [61]. However the flavonoids and phenolics present in significant amount in this plant are still unexplored. The preferential quantity of these compounds in the methanolic extract of Pant Aparna as revealed by the present study directed to focus towards the purification and characterization of potential compound from this variety.

## 4. Conclusion

Medicines derived from plants have made immense contribution towards the betterment of human health and act as a source of inspiration for novel drug compounds. From the above research it can be concluded that this plant has immense potential to be used in the area of pharmacology and as a prospective source of valuable drugs. Due to the presence of various compounds that are essential for good health, it can also be used to improve the health status of society. The extracts showed a significantly high antibacterial activity against the microorganisms. The data clearly depicts the presence of compounds used for treating various bacterial diseases, indicating its use in the traditional system of medicine since ancient times. Further, the broad spectrum activity of aqueous, methanol, and aqueous: ethanol extracts proves to be encouraging in the development of novel antimicrobial formulation in the near future.

A spectrum of compounds showing strong antibacterial, antioxidant, and anti-inflammatory activities was revealed by the GC-MS analysis of the methanolic extract of* Aegle marmelos*.

Antimicrobials derived from plants possess vast curative properties since they have fewer side effects as compared to synthetic antimicrobials drugs.* Aegle marmelos* is of utmost importance for ethnobotanical purposes, and it has been placed in the priority list of thirty-two medicinal plants by The National Medicinal Plants Board of Govt. of India [62]. The present study contributes to the current knowledge of presence of various phytochemical active compounds in 18 varieties/accessions of* Aegle marmelos* possessing significant broad spectrum antibacterial efficacy. Further fractionation and purification will elucidate the potential compound, which is a pressing need because of the upcoming resistance of the currently available antibiotics.

## Figures and Tables

**Table 1:** Qualitative analysis of phytochemicals in aqueous and methanol extract of different varieties/accessions of Bael (*Aegle marmelos*) leaves.

Diff. Bael variety/accessions	^ a^TA	^ b^PHL	^ c^SAP	^ d^TER	^ e^FLA	^ f^CAR	^ g^ANTH	^ h^CAR	^ i^RED	^ j^ALK
NB-17	+	−	+	+	+	+	−	+	+	+
Pant Aparna	+	−	+	+	+	+	−	+	+	+
NB-9	+	−	+	+	+	+	−	+	+	+
NB-5	+	−	+	+	+	+	−	+	+	+
AM-4	+	−	+	+	+	+	−	+	+	+
NB-7	+	−	+	+	+	+	−	+	+	+
AM-7	+	−	+	+	+	+	−	+	+	+
AM-3	+	−	+	+	+	+	−	+	+	+
NB-1	+	−	+	+	+	+	−	+	+	+
Kaghzi	+	−	+	+	+	+	−	+	+	+
NB-4	+	−	+	+	+	+	−	+	+	+
NB-16	+	−	+	+	+	+	−	+	+	+
P.Sujana	+	−	+	+	+	+	−	+	+	+
P.Sujata	+	−	+	+	+	+	−	+	+	+
AM-1	+	−	+	+	+	+	−	+	+	+
AM-2	+	−	+	+	+	+	−	+	+	+
AM-6	+	−	+	+	+	+	−	+	+	+
AM-8	+	−	+	+	+	+	−	+	+	+

^a^TA: tannins; ^b^PHL: phlobatannins; ^c^SAP: saponins; ^d^TER: terpenoids; ^e^FLA: flavonoids; ^f^CAR: cardiac glycosides; ^g^ANTH: combined anthraquinones; ^h^CAR: carotenoids; ^i^RED: reducing sugar; ^j^ALK: alkanoids; +: present; −: absent.

**Table 2:** Quantitative estimation of phytochemicals (mg/g).

Different Bael variety/accessions	Alkaloids	Flavonoids	Phenols	Saponins	Tannins
NB-17	8.62 ± 0.10	26.2 ± 0.065	22.8 ± 0.004	7.65 ± 0.14	4.53 ± 0.15
Pant Aparna	16.08 ± 0.05**	63.9 ± 0.061**	29.4 ± 0.004**	11.98 ± 0.20	8.32 ± 0.40
NB-9	10.7 ± 0.15	19.8 ± 0.058	10.22 ± 0.032	5.36 ± 0.15	3.78 ± 0.35
NB-5	9.56 ± 0.10	23.2 ± 0.055	12.6 ± 0.005	6.57 ± 0.25	6.43 ± 0.05
AM-4	11.6 ± 0.14	23.9 ± 0.053	11.4 ± 0.007	4.23 ± 0.44	4.97 ± 0.10
NB-7	6.56 ± 0.25	18.5 ± 0.051	10.0 ± 0.005	4.65 ± 0.10	3.45 ± 0.25
AM-7	4.28 ± 0.30	39.1 ± 0.049	19.6 ± 0.045	13.40 ± 0.30	5.76 ± 0.35
AM-3	14.34 ± 0.20	28.9 ± 0.048	17.78 ± 0.079	12.76 ± 0.20	7.24 ± 0.15
NB-1	3.78 ± 0.15	10.4 ± 0.047	5.8 ± 0.085	4.21 ± 0.45	3.20 ± 0.25
Kaghzi	5.94 ± 0.15	17.4 ± 0.046	11.4 ± 0.004	5.85 ± 0.35	5.31 ± 0.18
NB-4	10.57 ± 0.30	12.4 ± 0.043	9.6 ± 0.004	7.50 ± 0.24	3.60 ± 0.42
NB-16	5.80 ± 0.25	10.4 ± 0.007	7.6 ± 0.007	3.20 ± 0.15	4.35 ± 0.36
P.Sujana	7.45 ± 0.45	16.4 ± 0.070	15.82 ± 0.007	6.02 ± 0.30	6.62 ± 0.28
P.Sujata	11.98 ± 0.25	23.9 ± 0.088	13.64 ± 0.008	8.53 ± 0.25	6.43 ± 0.08
AM-1	12.79 ± 0.20	23.5 ± 0.225	15.22 ± 0.082	7.89 ± 0.55	4.05 ± 0.24
AM-2	8.43 ± 0.35	12.4 ± 0.045	8.6 ± 0.084	5.9 ± 0.34	5.32 ± 0.31
AM-6	15.03 ± 0.40	26.9 ± 0.108	20.22 ± 0.010	10.94 ± 0.28	10.54 ± 0.05
AM-8	13.36 ± 0.25	26.6 ± 0.078	18.38 ± 0.078	9.25 ± 0.55	7.29 ± 0.18

**refers to significant values (*P* < 0.01).

**Table 3:** Zone of Inhibition by aqueous extract.

Aqueous extract								
Conc. (mg/mL)	10	15	20	25	30	40	Standard	Control

Name of bacteria	Zone of inhibition (mm)						Ampicillin (10 mcg/disc)	DMSO

*K. pneumoniae *	7.33 ± 0.03	8.33 ± 0.03	8.5 ± 0.02	6.5 ± 0.02	9.83 ± 0.04	10.6 ± 0.03	21.3 ± 0.088	0
*B. cereus *	7.33 ± 0.03	8.33 ± 0.03	9.00	9.00	9.00 ± 0.05	9.33 ± 0.05**	10.3 ± 0.088	0
*E. aerogenes *	8.00	8.00	8.33 ± 0.03	9.00	9.00	9.00**	11.3 ± 0.088	0
*S. aureus *	8.66 ± 0.03	9.66 ± 0.03	10.6 ± 0.03	11.6 ± 0.03	12.6 ± 0.03	13.0	40.6 ± 0.120	0
*S. epidermidis *	9.66 ± 0.03	11.0	12.0	13.0	13.6 ± 0.03	14.3 ± 0.06*	20.6 ± 0.145	0

Values are expressed as mean ± SEM and analyzed by one-way analysis of variance (ANOVA) followed by Dennett's *t* test; *P < 0.05; **P < 0.01.

**Table 4:** Zone of inhibition by methanol extract.

Methanol extract								
Conc. (mg/mL)	10	15	20	25	30	40	Standard	Control

Name of bacteria	Zone of inhibition (mm)						Ampicillin (10 mcg/disc)	DMSO

*K. pneumoniae *	9.3 ± 0.03	9.6 ± 0.03	10.6 ± 0.03	11.3 ± 0.03	11.0 ± 0.05	11.0 ± 0.05*	21.3 ± 0.088	0
*B. cereus *	8.6 ± 0.03	10.0	10.6 ± 0.03	11.3 ± 0.03	10.6 ± 0.03	12.0 ± 0.05**	10.3 ± 0.088	0
*E. aerogenes *	—	—	—	—	—	—	11.3 ± 0.088	0
*S. aureus *	10.0	11.0	11.0	12.3 ± 0.03	12.0	13.0	40.6 ± 0.120	0
*S. epidermidis *	8.0 ± 0.05	9.3 ± 0.06	10.0	10.3 ± 0.03	11.0	12.0*	20.6 ± 0.145	0

Values are expressed as mean ± SEM and analyzed by one-way analysis of variance (ANOVA) followed by Dennett's *t* test; *P < 0.05; **P < 0.01.

**Table 5:** Zone of inhibition by aqueous: ethanol extract.

Aqueous: ethanol				
Conc. (mg/mL)	40	80	120	160

Name of bacteria	Zone of inhibition (mm)			

*K. pneumonia *	9.0 ± 0.05	11.0 ± 0.05	12.3 ± 0.03	14.6 ± 0.06*
*B. cereus *	9.0	10.0	11.0	11.3 ± 0.03
*E. aerogenes *	10.0	15.0	16.0	18.3 ± 0.03**
*S. aureus *	10.3 ± 0.03	11.6 ± 0.03	12.0	13.0
*S. epidermidis *	10.3 ± 0.03	12.3 ± 0.03	13.3 ± 0.06	14.6 ± 0.03*

Values are expressed as mean ± SEM and analyzed by one-way analysis of variance (ANOVA) followed by Dennett's *t* test; *P < 0.05; **P < 0.01. The standard antibiotic and control are the same as that of aqueous and methanolic extract.

**Table 6:** Activity of the phytocomponents identified from methanolic leaf extract of *Aegle marmelos*.

S. no.	R.T	Compound name	Molecular formula	MW	Peak area %	Compound nature	**Activity
1	7.178	4H-Pyran-4-one, 2,3-dihydro-3,5-dihydroxy-6-methyl-	C_6_H_8_O_4 _	144	1.11	Flavonoid fraction	Antimicrobial, anti-inflammatory, antiproliferative
2	6.555	1-Butanol, 3-methyl-, acetate	C_7_H_14_O_2_	130	5.32	Alcoholic compound	Antimicrobial
3	12.09	2,3 Dioxabicyclo[2.2.2]oct-5-ene, 1-methyl-4-(1-methylethyl)-(Limonene dioxide 1)	C_10_H_16_O_2 _	168	0.53	Terpene	antimicrobial activity
4	13.385	Bicyclo[3.1.1]heptane-2,3-diol, 2,6,6-trimethyl (2,3-Pinanediol)	C_10_H_18_O_2 _	170	0.97	Terpene	antimicrobial activity
4	13.762	2-Cyclohexen-1-one, 4-hydroxy-3-methyl-6-(1-methylethyl)-	C_10_H_16_O_2 _	168	0.63		antibacterial
5	15.203	1-Dodecanol	C_12_H_26_O	186	4.83	long-chain fatty alcohol	antibacterial
6	16.143	Phenol, 2,6-bis(1,1-dimethylethyl)-4-methyl (BHT)	C_15_H_24_O	220	0.87		Antimicrobial, antioxidant activity
7	16.457	Benzoic acid, 4-ethoxy-, ethyl ester	C_11_H_14_O_3_	194	0.33	Aromatic acid ester	Antimicrobial Preservative
8	18.101	2-Propanol, 1,1′-[(1-methyl-1,2-ethanediyl)bis(oxy)]bis-(Tripropylene glycol)	C_9_H_20_O_4_	192	0.87		Antimicrobial activity
9	20.189	1-Tetradecanol, acrylate	C_17_H_32_O_2_	268	1.46	Fatty acid esters	Anti-inflammatory, antimicrobial
10	20.523	1,3,4,5-Tetrahydroxy-cyclohexanecarboxylic acid (Quinic acid)	C_7_H_12_O_6_	192	0.39	Aromatic acid	antimicrobial activity, anti-inflammatory
11	21.839	Tetradecanoic acid (Myristic acid)	C_14_H_28_O_2_	228	2.00	Fatty acid	Antifungal, Antioxidant, cancer preventive, nematicide, hypercholesterolemic, Lubricant
12	22.070	2(4H)-Benzofuranone 5,6,7,7a-tetrahydro-6-hydroxy-4,4,7a-trimethyl	C_11_H_16_O_3_	196	0.26	Triterpene	antimicrobial
13	22.262	1-Heptadecanol (1-Eicosanol)	C_17_H_36_O	256	0.33	Aliphatic alcohol	Antimalarial, antifungal, Antioxidant
14	22.409	1,3-Cyclohexadiene, 2-methyl-5-(1-methylethyl)-(1-Phellandrene)	C_10_H_16 _	136	0.23	Monoterpene	Antibacterial
15	22.736	1,6-Octadiene, 7-methyl-3-methylene (beta.-myrcene)	C_10_H_16 _	136	0.13	Monoterpene	Antibacterial
16	23.404	2-Propenoic acid, 3-(4-hydroxy-3-methoxyphenyl)-, methyl ester (Cinnamic acid, 4-hydroxy-3-methoxy-, methyl ester )	C_11_H_12_O_4_	208	0.66	Aromatic methyl esters	Antimicrobial, antioxidant, antiviral
17	23.819	Pentadecanoic acid	C_15_H_30_O_2 _	242	0.08	Fatty acid	Antibacterial
18	24.897	3,7,11,15-Tetramethyl-2-hexadecen-1-ol (Phytol)	C_20_H_40_O	296	0.32	Diterpene	Antimicrobial, anticancer, anti-inflammatory, anti-diuretic,
19	24.986	hexadecanoic acid, methyl ester (Palmitic acid methyl ester)	C_17_H_34_O_2_	270	0.72	Fatty acid methyl ester	Antioxidant, hypocholesterolemic nematicide, pesticide, antiandrogenic flavor, hemolytic, 5-Alpha reductase inhibitor
20	24.951	Pentadecanoic acid	C_15_H_30_O_2_	242	7.99	Saturated fatty acid	Antimicrobial
21	27.266	9-Octadecenoic acid	C_18_H_34_O_2_	282	0.31	Unsaturated fatty acid	antibacterial
22	27.664	Heptadecanoic acid	C_17_H_34_O_2_	270	0.39	Saturated fatty acid	Antimicrobial
23	28.298	9,12,15-Octadecatrienoic acid, methyl ester (Linolenic acid, methyl ester)	C_19_H_32_O_2_	292	1.07	Fatty acid methyl ester	Antibacterial and anticandidal Antiinflammatory, hypocholesterolemic, cancer preventive, hepatoprotective, nematicide, insectifuge antihistaminic, antiarthritic, anticoronary, antieczemic antiacne, 5-Alpha reductase inhibitor Antiandrogenic
24	28.554	2-Hexadecen-1-ol, 3,7,11,15-tetramethyl (Phytol isomer)	C_20_H_40_O	296	6.37	Diterpene	Antimicrobial Anti-inflammatory Anticancer Diuretic
25	29.227	Cis-9-Hexadecenal	C_16_H_30_O	238	11.40	Aldehyde	Antimicrobial
26	29.573	Octadecanoic acid (Stearic acid)	C_18_H_36_O_2_	284	4.06	Fatty acid	Antimicrobial
27	41.240	Benzene, 1,2-dimethoxy-4-[[(4 methylphenyl)sulfonyl]methyl	C_16_H_18_O_4_S	306	10.76	Aromatic sulfur compound	Antimicrobial
28	44.155	Cholest-5-en-3-ol (3.beta.)-	C_27_H_46_O	386	0.24	Steroidal	Antibacterial
29	45.238	Ergost-5-en-3-ol, (3.beta.)-	C_27_H_46_O	386	0.50	Steroidal	Antimicrobial, anti-inflammatory effects
30	45.509	Stigmasta-5,22-dien-3-ol	C_29_H_48_O	412	0.63	Steroidal	Antioxidant, antibacterial activity, antiinflammatory, antiarthritic antiasthma, diuretic
31	46.038	Stigmast-5-en-3-ol, (3.beta.)-	C_29_H_50_O	414	3.08	Steroidal	Antimicrobial antioxidant antiinflammatory antiarthritic antiasthma diuretic
32	47.033	Vitamin E	C_29_H_50_O_2_	430	0.58		Antioxidant and Antimicrobial activity Analgesic, Antidiabatic Antiinflammatory, Antidermatitic, Antileukemic, Antitumor, Anticancer, Hepatoprotective, Antispasmodic
33	44.454	alpha.-Tocopherol	C_29_H_50_O_2_	430	0.26		Anti-inflammatory, antioxidant, antimicrobial, radical scavenging, antispasmodic

**Source: Dr. Duke's: Phytochemical and Ethnobotanical Databases.
